# A nutritional intervention for moderate altitude endurance preparation: A case report

**DOI:** 10.1080/15502783.2022.2140596

**Published:** 2022-11-16

**Authors:** Juan J. Guerrero-Pinzón, Juan M.A. Alcantara, Gustavo García-Buendia, Sol Mochón-Benguigui, Mauricio Ramírez-Maldonado, Jonatan R. Ruiz, Lucas Jurado-Fasoli

**Affiliations:** a PROmoting FITness and Health through Physical Activity Research Group (PROFITH), Sport and Health University Research Institute (iMUDS), University of Granada, Department of Physical Education and Sports, Faculty of Sport Sciences, Granada, Spain; b University of Granada, Department Physical Education and Sports, Faculty of Sport Sciences, Granada, Spain; c University of Granada, Department of Physiology. Faculty of Medicine, Granada, Spain; d Instituto de Investigación Biosanitaria, ibs.Granada, Granada, Spain

**Keywords:** Altitude, mountain, ski, trail running, nutritional intervention, dietary supplements, ergogenic aids

## Abstract

**Background:**

Moderate altitudes carry physiological and metabolic changes that can dampen exercise performance. Fortunately, these changes can be modulated by an optimal nutritional intervention. This case study represents the first nutritional intervention of a moderate altitude athlete. These results may help to establish well-designed nutritional guidelines for moderate altitude sports athletes.

**Case presentation:**

This case study examined the effects of a 11-week nutritional intervention on body composition, muscle strength, cardiorespiratory fitness, resting and exercise nutrient oxidation, and subjective sleep quality, in a male high-level moderate altitude athlete with a very light non-exercise activity thermogenesis. During the 11-week of nutritional intervention, 2800-3500 kcal/day, 6.8-8.9 g/kg/day of carbohydrates, 1.2-1.7 g/kg/day of protein, and 1-2.5 g/kg/day of fat were prescribed. Different specific considerations were also included, such as: iron supplementation, antioxidants increment in different phases, and ergogenic aids (i.e. creatine and beta-alanine). Our results demonstrated a decrease in adiposity and an increase in fat-free mass. In parallel, the athlete improved muscle strength, and therefore endurance adaptations after a maximal effort test (i.e. enhancement of the heart rate recovery). After the intervention, the athlete not only increased the carbohydrate oxidation during exercise and resting conditions but also improved his subjective sleep quality.

**Conclusions:**

Our results suggest that a nutritional intervention based on the endurance nutritional recommendations and adapted to the altitude physiological peculiarities can induce body re-composition, improve physiological adaptations to effort, and upgrade the substrate oxidation in a moderate altitude high-level athletes.

## 1. Background

Nutrition in altitude sports, specifically in moderate altitudes (~1500–3000 m above sea level), is an emerging topic due to the environmental and physiological stressors of these disciplines which directly influence exercise performance [1,2]. In line with this, at moderate altitudes, there is an ~5% decrease in VO_2peak_ [3] and appetite sensations. Furthermore, moderate altitudes increase the resting metabolic rate (RMR), glycogen use, and oxidative stress [2]. The above-mentioned physiological and metabolic changes during altitude could be potentially modulated by an optimal nutritional intervention [2] through an increase in the energy intake (EI), carbohydrates (CHO), antioxidants, and/or iron [2,4]. Therefore, the impact of nutrition and supplementation on exercise performance at moderate altitudes is of scientific interest.

Unfortunately, the current nutritional recommendations in altitude conditions are based on studies conducted at high to extreme altitudes (>3000 m), which may not be comparable with sports performed at moderate altitudes [5]. Nowadays, the nutritional recommendations for moderate altitude sports are similar to those for sports performed at sea level [6]. Therefore, the establishment of well-designed nutritional recommendations focused on moderate altitude sports is of scientific interest. Altogether, this case study examined the effects of a 11-week nutritional intervention on body composition, muscle strength, cardiorespiratory fitness, resting and exercise nutrient oxidation, and subjective sleep quality, in a male high-level moderate altitude athlete (≈2000-3400 m above sea level; Spanish national level).

### Case presentation

The study athlete was a male, 23-year-old, high-level moderate altitude athlete in the disciplines of trail running and ski mountaineering (Skimo). The athlete had an initial weight of 56 kg, with a height of 166.5 cm, a body fat percentage of 16.6%, and a cardiorespiratory fitness of 76.5 ml/kg/min. The athlete had more than 5 years of altitude training experience and in altitude competitions (from 1300 to 3400 m). Initially, the athlete was evaluated by a medical researcher with a precise anamnesis, a careful clinical examination, and routine tests. Furthermore, the study athlete was not on any prescribed medication, was a nonsmoker, and had not followed any nutritional intervention or dietary advice prior to his inclusion in the current study.

Briefly, the study athlete trained between 1 and 5 h/day, with a frequency of 5-6 days/week, and a total volume of 10-15 h/week in Sierra Nevada, Granada (≈2000-3400 m above sea level) involving both trail running and skimo disciplines. During the complete nutritional intervention, the athlete performed his autonomous (unsupervised) training sessions without experiencing significant changes in his training routine. Nevertheless, all training sessions were weekly registered in a training diary during the 11 weeks of the intervention (data not shown).

Before the intervention, an exhaustive nutritional anamnesis was performed by a registered dietitian to assess potential nutritional allergies, intolerances, preferences, lifestyle, training schedules, etc. Of note, the study participant had an EI of ≈4100 kcal/day, with a high intake of protein (2 g/kg/day), and fat (2 g/kg/day), and a low intake of CHO (4 g/kg/day) and fiber (13 g/day). The main food sources of his diet were ultra-processed foods (e.g. commercial pizza), and fried foods (e.g. French fries) providing the 15-20% of his daily energy intake.

**Figure 1.**
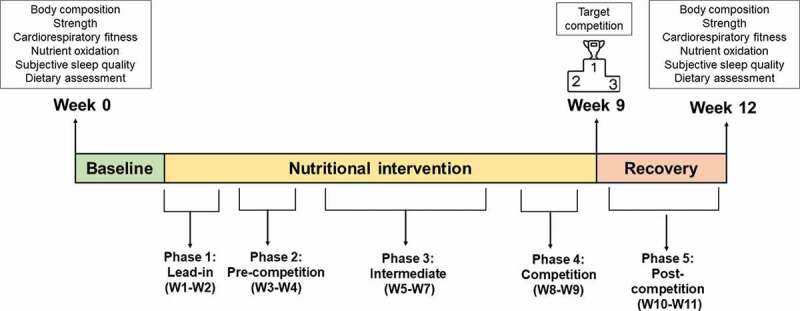
**Study design**. *Abbreviation*: W, week.

The design of the current study is shown in Figure 1. The nutritional intervention had a total duration of 11 weeks considering the athlete’s competition goals (Spanish Championship of Vertical Kilometer and Spanish Championship of Trail). During the whole intervention, the nutritional intake and ergogenic aids/supplementation were prescribed by a registered dietitian. All dependent outcomes were assessed before starting the nutritional intervention and after the 11 weeks of nutritional intervention, in the same research center (Granada, Spain; ≈738 m above the sea level) and following exactly identical procedures.

The participant provided written informed consent prior to participation in the current study and read the current manuscript before submission. The research procedures were approved by the Ethics Committee on Human Research at the University of Granada (1996/CEIH/2021) and were in accordance with the CARE guidelines.

### Assessments of dependent outcomes

All the assessments were carried out following standardized protocols during the weeks 0 and 12 (see Figure 1). Extended description and information can be found in the supplementary material.

First, the participant attended to the research center (Sport and Health University Research Institute, iMUDS) after a rest day early in the morning after a 10 h overnight fast, avoiding stimulants 12 h before and drugs 24 h before, with light clothing and without shoes. The RMR was assessed during 30 min using a metabolic cart (Omnical, Maastricht Instruments, Maastricht, The Netherlands) and following current recommendations [7]. Using the measured gas exchange, both exercise and resting nutrient oxidation were estimated using the Frayn equations [8] for CHO and fat oxidations (CHOox and FATox respectively), and the Weir equation [9] was utilized for estimating the RMR. For all equations, the excreted urinary nitrogen was considered to be 0. Then, body composition was measured by dual-energy X-ray absorptiometry using a Discovery Wi device (Hologic Inc., Bedford, MA, USA) and analyzed with its specific software (APEX version 4.0.2). The equipment was daily calibrated with a spine phantom for correction of sources of error (coefficient of variation for whole body composition of ≈0.5% [10]).

Cardiorespiratory fitness (VO_2max_) was assessed on the same day in the evening through a maximal incremental test on a treadmill (H/P/Cosmos Sports & Medical GmbH, Nussdorf-Traunstein, Germany) following a previously validated protocol in trail runners [11]. The gas exchange (Ultima CardiO2, Medgraphics Corp., MN, USA) and the heart rate (HR) (Polar RS800CX, Kempele, Finland) were continuously registered during the maximal incremental test. Further, from the assessed gas exchange the ventilatory equivalents for oxygen and carbon dioxide (VE/VO_2_ and VE/VCO_2_ respectively) were estimated and the ventilatory thresholds 1 and 2 (VT_1_ and VT_2_ respectively) determined. We considered that VT_1_ was achieved at that moment in which an increase in VE/VO_2_ was observed with no increase in VE/VCO_2_ and deviation from linearity of minute ventilation (VE) [12]. VT_2_ was considered as that moment in which VE/VO_2_ and VE/VCO_2_ concomitantly increased [12]. Both, VT_1_ and VT_2_ were determined by two independent observers (LJF and JMA).

48-h apart from the VO_2max_ assessments, muscle strength parameters (one-repetition maximum and power) were assessed with a DynaSystem Research Functional Dynamometer (SYMOTECH, Granada, Spain) following standardized protocols to evaluate one-step (lower body), row (upper body), and mid-thigh pull (lower body) strength parameters [13].

Lastly, subjective sleep quality was evaluated with the Pittsburgh Sleep Quality Index (PSQI; higher scores indicate worse sleep quality) [14].

### Nutritional intervention

The nutritional intervention was divided into five phases in accordance with the nutritional, recovery, and competition objectives. The nutritional intervention was built using the Dietopro® software, which employs the United States Department of Agriculture (USDA database). Ergogenic aids and dietary supplements required for the nutritional intervention were provided by HSN® (Harrison Sport Nutrition (HSN) Store, Granada, Spain). Table 1 shows a summary of the intervention; nonetheless, an extensive description can be found in the supplementary material. Of note, the nutritional intervention was based on the current nutrition guidelines for endurance athletes [15] adapted specifically for moderate altitude physiological considerations [2,15–19]. The specific considerations included were: i) iron supplementation; ii) CHO increments during exercise; iii) antioxidant increments in different phases; and iv) ergogenic aids (i.e. creatine and beta-alanine).

**Table 1.** Summary of the nutritional intervention.

	Phase 1: lead-in		Phase 2: pre-competition		Phase 3: intermediate			Phase 4: competition		Phase 5: post-competition	
	W1	W2	W3	W4	W5	W6	W7	W8	W9	W10	W11
Energy intake (kcal/day)	2800-3000	2900-3100	3000-3200	3000-3200	3250-3500	3100-3500	2900-3300	2600-3000	2700-3500	2300-3000	2300 – 2800
CHO intake (g/kg/day)	7	7.7	8.9	7	6.4	6.8	6.8	7.3	7.3	6.8	7.3
Protein intake (g/kg/day)	1.2	1.2	1.7	1.5	1.7	1.7	1.6	1.5	1.5	1.2	1.3
Fat intake (g/kg/day)	1.2	1	1.2	1.4	2	2.5	1.5	1.4	1	1.3	1.4
Antioxidants intake	→	→	→	→	↓	↓	↓	↑	↑	↑	↑
Iron intake	→	→	→	+	+	→	→	+	+	→	→
Supplementation and ergogenic aids	-	Caffeine, gels	Evocarbs, gels, caffeine, creatine, beta-alanine	Evocarbs, gels, caffeine, creatine, beta-alanine, Evolytes	Evocarbs, gels, caffeine, creatine, beta-alanine, Evolytes	Evocarbs, gels, caffeine, creatine, beta-alanine, Evolytes	Evocarbs, gels, caffeine, creatine, beta-alanine, Evolytes	Evocarbs, gels, caffeine, creatine, beta-alanine, Evolytes	Evocarbs, gels, caffeine, beta-alanine, Evolytes	Evocarbs, gels, creatine, beta-alanine	Evocarbs, gels, creatine
Peculiarities	Nutritional education	Nutritional education + Intra-nutrition	Nutrition for competition	Nutrition for competition	Gut training, ↑CHO	Energy and CHO periodization, ↑calories	Energy and macronutrient readjustments	Competition preparation	Target competition	Energy and liquid reposition	Energy and liquid reposition
Competition	No	Yes	Yes	Yes	No	No	No	No	Yes	No	No

*Abbreviations*: →, not modify; ↓, decrease; ↑, increase; +, iron supplementation; CHO, carbohydrates; W, week. All dietary supplements and ergogenic aids were provided by Harrison Sport Nutrition (HSN®) Store, Granada, Spain. See supplementary table S1 for the specific nutritional information of the dietary supplements.

Total energy expenditure (TEE) was estimated from the RMR values obtained in the baseline assessment (i.e. 1829 kcal/day), plus 10% of the thermic effect of food (i.e. 180 kcal/day), and the energy expenditure derived from the physical activity of each phase (i.e. 550-750 kcal/h of training), and the non-exercise activity thermogenesis (very light; 2000-3000 steps/day). Additionally, to restructure the nutritional intervention, a weekly body weight record was performed with an electronic weight scale (Tanita BC-730, Tanita Corp, Japan) under standardized conditions (e.g. controlled fasting timing, hydration level). Both, the energy and macronutrients intake reported in the different phases, are actual intakes.

**Phase 1, lead-in** (Weeks 1-2): The initial intervention was focused on ‘nutritional education’ to increase the intake of healthy food, and decrease the intake of ultra-processed food and fried preparations. In parallel, the energy and macronutrient requirements were prescribed accordingly to the nutritional recommendations in endurance athletes’ (2800-3100 kcal; CHO: 7-7.7 g/kg/day; Protein: 1.2 g/kg/day of high biological value; Fat: 1-1.2 g/kg/day) [15,16,18,19].

A 3.6 mg of caffeine/kg body weight (or 4.3 mg of caffeine/kg of fat-free mass; FFM) was prescribed during the second week of the Phase 1 (30-45 min prior to the training session, 1-2 times per week; Anhydrous Caffeine Tablets, HSN®, Granada, Spain) [20,21]. Furthermore, a progressive introduction of sports gels diluted in water was initiated to train the gut with the products to be used in the upcoming competitive events (Evoenergy sport gel with guarana and caffeine and Evoenergy sport gel without caffeine, HSN® Granada, Spain) [22]. During this phase, 30-45 g/h of CHO and 200-250 mg/h of Na^+^ were prescribed during the exercise training sessions which had a mean duration of 1:45-2 h [23]. Lastly, it was observed that the nutrition was prescribed accordingly as the last weekend of this phase the athlete had a competition event.

**Phase 2, pre-competition** (Weeks 3-4): The main objective of this phase was to optimize the physical performance of the competition events during both weekends. Therefore, due to the training accumulation of this phase and the decrease in the athlete’s body weight (1 kg) after phase 1, an increase in the EI (200-400 kcal/day) was carried out. The CHO and Protein (high biological value) intake prescriptions were gradually increased until an amount of 7-8.9 g/kg/day and 1.5-1.7 g/kg/day respectively, whereas the fat intake was maintained at 1.2-1.4 g/kg/day [15,16,18,19]. On the other hand, the day before the competition event, a 10 g/kg/day CHO intake was prescribed [15,16,18,19].

Furthermore, post-training intake of whey protein (15-30 g of Evowhey 2.0 [whey protein concentrate, WPC] HSN® Granada, Spain), 4 g/day of beta-alanine split in two separate intakes (Beta-alanine powder 100% RAW, HSN® Granada, Spain), and 5 g/day of creatine (Creatine monohydrate powder [100% Creapure®], HSN®, Granada, Spain) were prescribed to support the high-intensity and volume of the upcoming phases (Phase 3 and 4) and the athlete competition goals (week 9) [24–26]. Moreover, the diet during this phase had an iron content of 20-25 mg/day and was supplemented with a moderate dose of 50 mg of iron (Chelated Iron, HSN®, Granada, Spain) in the mornings accompanied with foods rich in vitamin C and avoiding foods rich in calcium, fiber, and caffeine [27,28]. The aim of the iron supplementation was to maintain an optimal iron balance to support both the hematological and non-hematological adaptations to altitude training and/or competitions and also to support training-induced iron losses [29]. Although no iron deficiency was reported, the participant had a low intake of iron food sources and reported a previous history of iron deficiency in the past year. Finally, foods rich in antioxidants were included in the daily diet of the athlete to heighten the recovery between competitions (i.e. berries shake, antioxidants infusions, and dark chocolate) [30], whereas caffeine was only included during competitions to periodize its supplementation and to avoid possible adaptations [31].

**Phase 3, intermediate** (Weeks 5-7): During this phase, there was no competition, and the athlete increased the intensity and volume of trail and Skimo training, and included resistance-training sessions. Consequently, an increase in the EI was prescribed (~250-500 kcal/day, +7-16%), until 3250-3500 kcal/day, maintaining the same proportion of CHO (6.4-6.8 g/kg/day) and protein (1.6-1.7 g/kg/day of high biological value protein), and slightly increasing the fat intake (2 g/kg/day) to easily achieve the energy requirements. The main objectives of this phase were to train the gut, and the periodization of EI and CHO according to the type of training sessions. In this sense, the EI and CHO intake prescriptions were augmented (~10%) those days when the athlete performed endurance training sessions (trail or Skimo; 3100-3500 kcal/day and 7.1-7.5 g/kg/day), and were diminished (~20-25%) when the athlete performed resistance training sessions or rested (2500-2700 kcal/day and 5-5.5 g/kg/day) [15,16,18,19].

To train the gut, moderate quantities of CHO (30-70 g/h) with high liquid volumes were prescribed during training sessions to increase the adaptations of the digestive system [22]. The training sessions had a duration between 2 h and 3.5 h. The supplementation with beta-alanine and creatine was required following the above-mentioned recommendations, whereas caffeine was only taken during high-volume/high-intensity training sessions only one or two times per week to maximize its ergogenic effects [31]. Iron supplementation stopped in the middle of this phase to start a rest period and avoid the possible saturation and adaptations [2,29,32]. Ultimately, foods rich in antioxidants were restricted during this phase with the aim of avoiding possible interference with the physiological adaptations of the training [33].

**Phase 4, competition** (Weeks 8-9): The objective of this phase was the double competition (Spanish Championship of Vertical Kilometer and Spanish Championship of Trail) preparation which were the main competition goals of the athlete.

During this phase, the EI was slightly lower than the previous phase (2900-3500 kcal/day). However, an increase in CHO intake was prescribed (5.4-8.5 g/kg/day) to enhance the recovery after the training sessions. Protein and fat intakes were slightly decreased (1.5 g/kg/day of high biological value and 1.4 g/kg/day). Energy and macronutrient requirements were achieved including foods and recipes previously used by the athlete with the aim to increase the adherence and avoid potential gastrointestinal problems.

Iron supplementation was re-introduced during this phase to avoid the anemia appearance due to the physiological stress, as well as food rich in antioxidants was re-included in the diet of the athlete to enhance the pre-competition recovery [30]. In addition, during the training sessions of this phase (1:45-2:45 h of duration), similar intra-training nutrition strategies (i.e. CHO and Na^+^) were used as mentioned above. In the end, beta-alanine supplementation was prescribed for these weeks, whereas creatine supplementation was removed the last week prior to the competition event to decrease the potential water retention and to re-adjust the body weight [34].

**Phase 5, post-competition** (Weeks 10-11): The objective of the last phase was to enhance the recovery after the training and competition periods. The EI and macronutrient prescription intake ebbed according to the lower training volume and intensities (i.e. to 2300-3000 kcal/day, 6.8 g/kg/day of CHO, 1.2 g/kg/day of protein, and 1.3 g/kg/day of fat). The intake of high-antioxidant foods and creatine supplementation were re-introduced to improve the recovery processes [30,35]. Eventually, the caffeine intake dwindled along the training sessions of this phase whereas the CHO intakes were maintained during the sessions (30-60 g/h of CHO in training session of 2 h-3 h of duration), and rehydration strategies were implemented [23].

### Results

**Figure 2.**
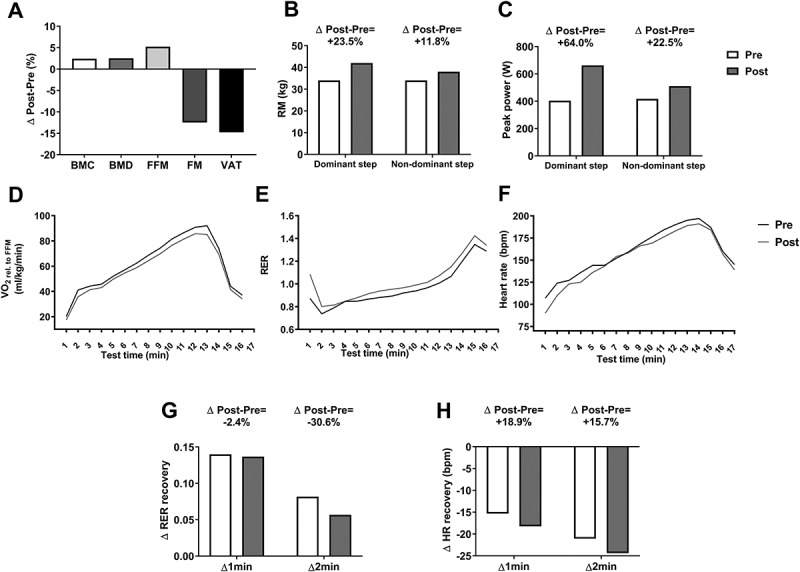
**Changes in body composition (Panel A), one-step muscle strength (Panels B-C), the dynamics of the outcomes derived from the maximal effort treadmill test (Panels D-F) and the RER and heart rate recovery after the maximal effort treadmill test (Panels G and H)**. Δ1 min was calculated as: Recovery value minute 2-value recovery minute 1; whereas Δ2 min was calculated as: value recovery minute 3-value recovery minute 1. *Abbreviations*: BMC, bone mineral content; BMD, bone mineral density; FFM, fat free mass; FM, fat mass; HR, heart rate; RER, respiratory exchange ratio; RM, one-repetition maximum; VAT, visceral adipose tissue; VO_2_, volume of oxygen consumption. Bpm: beats per minute; W, watts.

The study athlete's body weight escalated progressively during the nutritional intervention (week 0: 56.0 kg; week 4: 56.8 kg; week 8: 56.9 kg; and week 12: 57.3 kg). In line with this, a body re-composition was achieved once the nutritional intervention concluded (Figure 2A). The athlete ameliorated his bone parameters (bone mineral content: +2.4%; bone mineral density: +2.5%), increased his FFM content (+5.2%) and decreased his adiposity content (fat mass: −12.5%; visceral adipose tissue: −14.8%) (Figure 2A).

Additionally, the results showed that all the muscle strength parameters were improved after the nutritional intervention (Figure 2B, 2C**, and** Table 2). The study athlete not only increased his one-repetition maximum but also his power mean strength, peak strength of the one-step, row, and mid-thigh pull exercises (Figure 2B, 2C**, and** Table 2).

**Table 2.** Changes in muscle strength, cardiorespiratory fitness, nutrient oxidation, and subjective sleep quality.

	Week 0	Week 12	Change (%)
*Muscle strength*			
Row mean strength (kg)	54.1	55.7	+3.0
Row peak strength (kg)	61.3	62.3	+6.6
Mid-thigh pull mean strength (kg)	76.7	78.5	+2.3
Mid-thigh pull peak strength (kg)	87.3	91.0	+4.2
*Cardiorespiratory fitness*			
VO_2peak_ (ml/min)	4243	4127	−2.8
VO_2peak_ rel. to FFM (ml/kg/min)	92.1	85.2	−7.5
VT_1_ (% VO_2peak_)	65	69	+4
VT_2_ (% VO_2peak_)	82	82	0
Time until exhaustion (min)	14	14	0
*Resting gas exchange outcomes*			
RMR (kcal/day)	1829	1772	−3.1
CHOox (g/min)	0.10	0.25	+150
CHOox (%RMR)	31.5	81.2	+49.7
FATox (g/min)	0.09	0.03	−66.7
FATox (%RMR)	63.8	21.9	−41.9
*Subjective sleep quality*			
PSQI	3	2	−33.4

*Abbreviations*: RMR, resting metabolic rate; CHO, carbohydrates; CHOox, carbohydrate oxidation; FATox, fat oxidation; PSQI, Pittsburgh sleep quality index; VO_2peak_, peak of volume of oxygen consumption during the maximal effort test; VT_1_, first ventilatory threshold expressed as a percentage of the VO_2peak_; VT_2_, second ventilatory threshold expressed as a percentage of the VO_2peak_.

The duration of the maximal effort test (i.e. the time until exhaustion) after the 11-week nutritional intervention had the same duration as the baseline test (Table 2). Furthermore, VO_2peak_ in both absolute values (ml/min) and relative to FFM (ml/kg/min) values were lower in comparison to the athlete baseline values (Table 2). Similarly, over the test time, a lower VO_2_ dynamics relative to FFM was observed after the intervention period (Figure 2D). However, the VO_2_ dynamics in absolute values were similar between both assessments (**Figure S1A**). During the test, the athlete also displayed a slightly higher RER dynamics (Figure 2E) and a lower rating of perceived exertion (RPE) and HR values (**Figures S1C and** 2F respectively) when comparing the after nutritional intervention to the baseline values. We also observed a decrease in the change (Δ1 min and Δ2 min) of the RER in the recovery phase of the test (Figure 2G), and an increase in the change (Δ1 min and Δ2 min) of the HR, VO_2_, and VCO_2_ (Figure 2H**, S1D and S1E** respectively). As a result, the VT_1_ increased after the intervention whereas the VT_2_ did not change (Table 2).

After 11 weeks of nutritional intervention, the RMR of the athlete was slightly lower (Table 2), whereas the resting CHOox increased and the resting FATox decreased in both absolute values and expressed as a percentage of the RMR after the nutritional intervention (Table 2).

Finally, the subjective sleep quality improved by one point of the PSQI global score after the 11-week intervention (Table 2), specifically meliorating the diurnal dysfunction item.

## Discussion

The current study has designed an 11-week nutritional intervention based on the existing literature in endurance sports and includes different key points for the moderate altitude physiological demands [2]. Curiously, after the nutritional intervention the athlete experimented a body re-composition (reducing the adiposity content and increasing bone and FFM parameters). In line with this, the athlete showed higher levels of muscle strength production (e.g. one-repetition maximum, power mean strength), and more ideal endurance adaptations after a maximal effort test (e.g. enhancement of HR recovery after the intervention), experimented. Thus, the athlete displayed changes in nutrient oxidation during resting and exercise conditions (i.e. increasing the CHOox and reducing the FATox), and improved the subjective sleep quality. To our knowledge this is the first study performed so far of these characteristics, which may help to establish well-designed nutritional guidelines for moderate altitude sport athletes.

Altitude training/competitions events increase energy requirements mainly via the physiological stress induced by the altitude [2]. Changes in the energy requirements observed in altitude conditions could be driven by a shift in RMR and/or appetite regulation which could modify body composition and have a deleterious effect on physical performance [2]. Here, it is shown that a well-designed nutritional intervention could even produce a body re-composition increasing FFM and decreasing adiposity content in a high-level moderate altitude athlete. This body re-composition might have been driven by an adequate support of EI (i.e. reducing the risk of a low energy availability [36]), an adequate intake of CHO and protein [37], and the periodization of creatine supplementation and whey protein [24], together with the resistance exercise performed in phase 3. In agreement with this, both the FFM increments experimented by the athlete and the above-mentioned nutritional factors (i.e. energy availability, CHO, protein, and creatine) could have been the key determinants of the muscle strength improvements derived from the study athlete training. On the other hand, we observed a slight shift in the RMR (−3.1%), which could not be attributed to the nutritional intervention due to: i) the precision and reproducibility of the indirect calorimeters (≈4%) which could have influenced our assessments [38]; and ii) the physiological day-to-day variability of RMR in humans (3-8%) [39]. Both sources of bias, are close to the changes observed in the athlete. In parallel, an increase in CHOox and a reduction in FATox, in both conditions, resting and during exercise, were displayed after the intervention. This could be partially explained by the shift in the macronutrient intake induced by the intervention (i.e. a decrease in fat intake and an increase in CHO intake), since a higher intake of a concrete macronutrient, increases its oxidation as a primary energy source [40].

In altitude conditions, oxygen availability is a determinant in the maintenance of exercise, with the consequent decrease in VO_2_ [41]. In this sense, we observed a slight reduction in VO_2max_ [42], which could be partially explained by the changes in body composition of the study athlete, specifically the increase in FFM (+5.2%) [43]. This increment in FFM could also explain the improvements in the production of muscle strength, which is related [44] to a better cardiovascular and metabolic function [45].

Despite these changes, it is important to highlight that the HR and RPE during the maximal effort treadmill were lower after the intervention compared to the baseline values, which may indicate a better physiological adaptation to the same workload. In agreement with this, a more ideal RER and HR changes were achieved for the subject’s applications during the recovery phase of the test after 11-weeks, indicating a better recovery after the maximal effort test. Taking all these into account, these improvements in endurance adaptations might be a consequence of adequate energy availability derived from the increment in EI, an increment in the intake of CHO and its periodization, supplementation periodization (i.e. creatine and beta-alanine), hydration strategies, and different intra-training nutrition strategies (i.e. training the gut). All these strategies might have improved the recovery between training sessions, the training capacity, and delayed the fatigue during exercise with the final results in a better physiological adaptation to the effort.

During exercise, CHO provides fuel for the brain and skeletal muscle, with the delay in the appearance of fatigue and hypoglycemia [17]. In this sense, during exercise exerted in altitudes environments, there is a shift toward a greater CHO utilization [46]. Thus, to achieve an adequate exercise performance the capacity to oxidize CHO during endurance exercises becomes more important in altitude environments. Here, it was demonstrated that after 11-week of nutritional intervention the athlete raised the CHOox during a maximal effort test and in basal conditions, which could be explained by the increment in EI and CHO intake induced by the current intervention. This change is of practical importance due to the marked glycolytic character of the training/competition sessions (i.e. training sessions of 1-2 h and competitions of 15-45 min) of the athlete.

Lastly, it is worth mentioning that the study athlete had an adherence of 93% to the nutritional intervention and did not experience any secondary symptoms derived from the intervention. The athlete experienced the main side-effect of the beta-alanine supplementation (paresthesia), which decreased compliance with the intervention. There was a lack of supplementation for several days, but it was solved by splitting the total dosage into different ingestions. Since the beta-alanine supplementation was not completely missed, we think that no specific area of the intervention was affected. Thereby, our data suggest that an adequate nutritional intervention based on the endurance nutritional recommendations accompanied by supplement periodization (i.e. iron, caffeine, creatine, beta-alanine, and antioxidants), might be effective to increase the performance of the study athlete. However, it is necessary to take into account that the intervention was accompanied by the usual exercise training of the athlete, fact which could have partially influenced the results of the present study. Further clinical trials are needed to demonstrate the benefits of nutritional interventions in altitude sports. Despite the inherent limitations of the study, some light can be shed on the area of nutrition in altitudes, helping better to establish well-designed guidelines which can potentiate physical performance.

## Conclusions

There is a lack of evidence in the nutritional recommendations for altitude sports. This case study represents the first nutritional intervention accompanied by a supplementation periodization of a moderate altitude athlete. This study shows that an 11-week of nutritional intervention based on the endurance nutritional recommendations and adapted to the altitude physiological peculiarities (e.g. iron supplementation) can induce a body-re-composition, improve physiological adaptations to effort, and upgrade the substrate oxidation in a moderate altitude high-level athlete.

## Supplementary Material

Supplemental Material

## Data Availability

Please contact the authors for data requests.
