# Supplementary material for: A nutritional intervention for moderate altitude endurance preparation: A case report
Source: J Int Soc Sports Nutr. 2022 Nov 16;19(1):650–63. doi: 10.1080/15502783.2022.2140596 (PMC9673806; doi:10.1080/15502783.2022.2140596)
Supplement: Supplemental Material [file RSSN_A_2140596_SM2169.docx]

**SUPPLEMENTARY MATERIAL**

***Assessments of dependent outcomes***

*Anthropometric and body composition*

Body weight and height were measured using a model 799 scale and a stadiometer, respectively (both from Seca, Hamburg, Germany), without shoes and with light clothing. Body mass index (BMI) was calculated from weight and height (kg/m^2^).

Bone mineral content, bone mineral density, fat-free mass, fat mass, and visceral adipose tissue were assessed by dual-energy X-ray absorptiometry following the manufacturers’ recommendations.

*Strength parameters*

Strength parameters were assessed with DynaSystem Research Functional Dynamometer (SYMOTECH, Granada, Spain) following standardized protocols. A standardized warm-up was performed prior to the start of the tests, based on 5min of running, 5min of dynamic mobility, and dynamic stretching.

1. *One step test:* the test was performed for both the dominant and non-dominant steps. Firstly, a warm-up was performed with 10% of the body weight until completing 10 repetitions. Then, an incremental intra-set test was realized with increments of 4kg until failure, or inadequate exercise execution. One-repetition maximum (RM) and peak power (W) were obtained from both the dominant and non-dominant step tests.
2. *Row:* a warm-up with 10 kg and 10 repetitions was performed to determine the range of motion of the exercise. After that, the bar was placed in the middle of the range of motion. An isometric set during 6sec at 50% and at 80% of the subjective maximum isometric contraction was performed. Lastly, 2 isometric sets at 100% of 6sec of duration were performed, and the mean and peak strength were obtained (kg).
3. *Mid-thigh pull:* the athlete performed 3 muscle contractions of 6 sec at 25, 50, and 75% of the subjective maximum isometric contraction. Next, 3 sets of 6sec at maximum isometric contraction were performed, obtaining mean and peak strength (kg).

*Cardiorespiratory fitness*

To assess the cardiorespiratory fitness of the study athlete, an incremental maximum effort test was performed in a treadmill (H/P/Cosmos Sports & Medical GmbH, Nussdorf-Traunstein, Germany) under medical supervision. In this regard, the “Trail test” protocol (1), which was previously validated in trail runners, was used. Briefly, the test had a warm-up period of 3min at 8km/h (0% slope). Then, the test started at 10.0km/h (0.5% slope) with continuous increments of speed (0.5km/h per stage) and slope (1.0% per stage) every minute until volitional exhaustion. The test ended with a cool-down phase of 6.0km/h for 3min. During the whole protocol, the 6-20 Borg scale was applied to assess the rating of perceived exertion (RPE) at each stage and at exhaustion (during the last 15sec) (2). Heart rate (HR) was continuously recorded every 5sec (Polar RS800CX, Kempele, Finland).

During the whole test, a metabolic cart (Ultima CardiO2, Medgraphics Corp., MN, USA) was used to continuously record the gas exchange (volume of oxygen consumption [VO_2_] and volume of carbon dioxide production [VCO_2_]) using an oronasal face-mask (model 7400, Hans Rudolph Inc., Kansas City, MO, United States) equipped with a high-flow Prevent^TM^ metabolic flow sensor (Medgraphics Corp., MN, United States). Following the manufacturers’ recommendations, we performed a flow calibration using a 3-L calibration syringe before the test. Then, we calibrated the gas analyzers before the test using two standard gas concentrations(3). The Breeze Suite software (version 8.1.0.54 SP7, MGC Diagnostic, Medgraphics Corp., MN, USA) was used to average VO_2_ and VCO_2_ every 5 s.

The following criteria were applied for achieving VO_2max_: i) to reach a respiratory exchange ratio (RER) ≥ of 1.1; ii) a plateau in VO_2_ (change of <100 ml/min in the last 3 consecutive 10-s stages); and iii) a heart rate between 10 beats/min of the age-predicted maximal heart rate (209–0.73 ∗ age). Regarding the ventilatory threshold 1 and 2 (VT1 and VT2 respectively) determination, we followed the procedures proposed by Lucía et al. (4). In brief, to identify the VT1, we determined the gas exchange recording interval in which the VE∙VO_2_ and the P_ET_O_2_ increased concomitantly while the VE∙VCO_2_ remained unchanged (4). Regarding VT2, we determined the interval in which the VE∙VO_2_ and the VE∙VCO_2_ increased concomitantly while the P_ET_CO_2_ values decreased (4)Both, VT1 and VT2 were determined by two independent researchers (LJF and JMA).

*Resting nutrient oxidation*

Resting metabolic rate (RMR) and nutrient oxidation were assessed by indirect calorimetry following current recommendations (5). The athlete was instructed to arrive at the laboratory at 8 a.m. by car or by bus (avoiding any physical activity after waking up), and having fasted for at least 8 h. The study athlete was advised to avoid any moderate physical activity for the 24 h before the test day, or any vigorous-intensity exercise in the prior 48 h. The study athlete lay on a bed in a supine position, and was instructed to breathe normally and not to talk, fidget or sleep. The assessments were performed in the same quiet room, in well-controlled conditions of temperature and humidity (22-24ºC and 35-45% respectively).

Indirect calorimetry measurements were made over a 30 min period using the Omnical metabolic cart (Maastricht Instruments, Maastricht, The Netherlands). The resting VO_2_ and VCO_2_ data were downloaded every 5-sec, and the first and last 5-min data were discarded and the remaining 20-min data averaged. Then, the abbreviated Weir equation (6) was used to estimate EE (in kilocalories per day; kcal/day). Further, both fat and carbohydrate oxidations (FATox and CHOox, respectively) were estimated (in grams per day; g/day) using the equations proposed by Frayn (7). For RMR, FATox, and CHOox the nitrogen urinary excretion was considered to be 0.

*Subjective sleep quality*

The subjective sleep quality was assessed with the Pittsburgh sleep quality index. The PSQI is a self-report tool which consists of 19-item scale that provides 7 component scores (ranges 0-3): (i) subjective sleep quality (very good to very bad), (ii) sleep latency (≤ 15 minutes to > 60 minutes), (iii) sleep duration (≥ 7 hours to < 5 hours), (iv) sleep efficiency (≥ 85% to < 65% hours sleep/hours in bed), (v) sleep disturbances (not during the past month to ≥ 3 times per week), (vi) use of sleeping medications (none to ≥ 3 times a week), and (vii) daytime dysfunction (not a problem to a very big problem); with a total global score ranging from 0 to 21 (8). A PSQI global score higher than 5 indicates poor sleep quality (8).

***Nutritional intervention***

*Phase 1, lead-in:*

The nutrition intervention in the second week of this phase was designed taking into account the double competition even on the last weekend of this phase. The athlete competed on Saturday in the Regional Championship of Skimo Sprint, and in the Regional Championship of Skimo on Sunday (Sierra Nevada, Andalucia).

The Regional Championship of Skimo Sprint consisted of different rounds of Skimo sprints of 5min duration each round. A tablet of caffeine (200 mg) was prescribed 30-45 min prior to the first round and one sport gel without caffeine during the warmup (CHO 31 g of CHO each sport gel; Evoenergy without caffeine) (9). Between rounds, the athlete performed 2-3 mouth rinses with one sport gel without caffeine (31g of CHO; Evoenergy without caffeine) diluted in 250-500 ml of water. The sweat rate was calculated for the electrolytic reposition, whereas the energy and macronutrient reposition was achieved through his normal diet.

The Regional Championship of Skimo had an expected duration of 3-4 h. 30-45 min prior to the event, the athlete took a caffeine tablet (200mg). During the event, an amount of 45 g/h of carbohydrates was prescribed following his previously intra-nutrition training (9, 10). These carbohydrates were obtained from 5 gels (155 g of CHO) diluted in 500 ml of water, combining its intake with only water every 10-15 minutes. The recovery was achieved through the sweat rate and his normal diet was used to achieve the energy and macronutrient requirements previously reported.

*Phase 2, pre-competition:*

First week competition

The competition event was the National Championship of Skimo Race Teams in Candanchú (Huesca, Spain; 1500-2400m), with an expected duration of 3-4h. 3h prior to the event, a nutritional intake of 2/kg of CHO, 0.4g/kg of protein (24g), low-fat (9g), and low-fiber was prescribed using foods previously ingested by the athlete. This nutritional intake was accompanied by the ingestion of 250-500ml/h of water to achieve an adequate hydration status. In addition, 30-45min previous to the race, a Tablet of caffeine (200mg) and one sport gel without caffeine (31g of CHO; Evoenergy without caffeine) during the warm-up (10min previous to the start of the race) were prescribed.

During the first part of the race (1.30-2h), the athlete ingested one bottle with 35g of CHO (Evocarbs 2.0 HSN^®^) diluted in 500ml of water, whereas 5 sport gels (4 without caffeine and 1 with caffeine [75mg], 155g of CHO) was the nutritional intake for the last part of the race (last 1.30-2h), with a medium prescription of 63g/h of CHO and 180mg/h of Na+.

The post-competition recovery was achieved through his normal diet with a shake with 20g whey protein (Evowhey 2.0) and 50g of CHO (Evocarbs 2.0), contributing 1.2g/kg of CHO and 0.5g/kg of protein.

Second week competitions

During the second weekend of this phase, the athlete competed on Saturday in the Regional Championship of Skimo Chronoscaled, and in the Regional Championship of Skimo Race Teams on Sunday (Sierra Nevada, Andalucía; 2100-3000 m).

The Regional Championship of Skimo Chronoscaled had a duration of 45 min at high-intensity without the possibility of ingesting food during the event. Therefore, a Tablet of caffeine (200mg) 30-45 min prior the event, and one sport gel without caffeine (Evoenergy without caffeine) diluted in 250ml of water 10min prior to the event during the warm-up were prescribed.

For the Regional Championship of Skimo Race Teams, a breakfast was prescribed 3-4 h prior de event with 2.1 g/kg of CHO and 0.5 g/kg of protein, low-fat and low-fiber. 30-45 min prior to the event, a Tablet of caffeine (200mg) was ingested with 500 ml of water and one tablet of mineral salts (Evolytes, HSN^®^). During the race, 35 g of CHO (Evocarbs) diluted in 500 ml of water was prescribed for the first part (2h), whereas 4 sports gels (2 with caffeine and 2 without), with 1 g of salt were diluted in 500 ml of water for the last part (2 h). The mean nutritional intake was 45-53g/h of CHO, 350 mg of caffeine, 343 mg/h of Na^+^ and 74 mg of K, with a Na^+^:K ratio of 4:1 (9). The post-competition nutrition was based on whey protein, CHO (Evocarbs) and with the athlete’s normal diet until achieving 2.5 g/kg of CHO and 0.9 g/kg of protein (9).

*Phase 4, competition:*

Spanish Championship of Vertical Kilometer

The event had an expected duration of 40-45 min in continuous uphill until complete +1000m of unevenness (Sierra Magina, Jaen; ≈2200 m). The day before the event, a CHO load was prescribed (10 g/kg/day), decreasing protein intake (1.3 g/kg/day of lean protein), fat (0.7 g/kg/day), and fiber. Iron supplementation was stopped to include different foods that the athlete used to eat which could interfere with its absorption (i.e., dairy products).

The breakfast of the competition day was high in CHO (2.8 g/kg), moderate in protein (0.5 g/kg), and low-fat (9 g). 1h30min prior to the event, a hypotonic drink with 5.2% of CHO was prescribed to improve the hydration and the glycogen stores (500ml water, 30g of Evocarbs, and 0.4 g of salt; 26 g of CHO and 390mg of Na^+^ per 500ml). Next, 30-45 min prior to the event, a tablet of caffeine (200mg) was prescribed, whereas a sport gel without caffeine with 200-400 ml of water was included 10min prior to the event during the warm-up.

Lastly, a recovery shake was elaborated with whey protein, Evocarbs, and water (35 g CHO and 24.3 g of protein). Following this initial intake, the normal diet was continued to achieve the energy and macronutrient requirements until the following competition.

Spanish Championship of Trail

The event had an expected duration of 4 h to finish 27.5 km of trail running with 2 refreshment points at 5 km and 15 km (Sierra Magina, Jaen; ≈220 0m). Before the event, the profile of the event, the climatology, the race rhythm among others factors were analysed. After completing the race of the Spanish Championship of Vertical Kilometer, another CHO load was prescribed, to achieve 11.3 g/kg of CHO, maintaining the protein and fat intakes (1.3 g/kg and 0.7 g/kg).

The breakfast prior to the event was high in CHO (2.8 g/kg), moderate in protein (0.5 g/kg), and low-fat (9 g). The same hypotonic drink as the previous day was prescribed 1h30min prior to the event. In addition, a tablet of caffeine (200 mg) and a tablet of mineral salts (250mg Na^+^ and 60 mg K) were prescribed 30-45 min prior to the event.

During the first part of the race (0-1 h), the athlete ingested 500ml of water and 40 g of Evocarbs diluted in 500 ml of water (35g of CHO). During the second part (1-1h30min; 5km-15km), 500 ml of water and 3 sports gels (2 with and 1 without caffeine) diluted in 500ml of water were prescribed. During the last part, (1h; 15 km-27.5 km), the athlete ingested 500 ml of water and 500 ml of water with 1g of salt, and 3 sports gels (1 with and 2 without caffeine) diluted. In summary, 236 g of CHO and 3 L of water were ingested, involving 70-73 g/h of CHO, 524 mg/L of Na^+^ ,146 mg/L of K (Na^+^:K ratio 3.6:1), and 425 mg of caffeine (4.25 mg/kg FFM of caffeine every 2h) (9).

A recovery shake based on whey protein, Evocarbs, and water was prescribed for the post-competition intake. The habitual diet was continued until achieving the recovery requirements (2.2 g/kg of CHO in the first solid intake, 2 g/kg/day of protein, and water).

**REFERENCES**

1. Scheer V, Ramme K, Reinsberger C, & Heitkamp HC. VO 2 max Testing in Trail Runners: Is There a Specific Exercise Test Protocol? *International Journal of Sports Medicine* 2018 **39** 456–461. (doi:10.1055/a-0577-4851)

2. Borg GA. Psychophysical bases of perceived exertion. *Medicine and science in sports and exercise* 1982 **14** 377–381.

3. Alcantara JMA, Sanchez-Delgado G, Martinez-Tellez B, Merchan-Ramirez E, Labayen I, & Ruiz JR. Congruent validity and inter-day reliability of two breath by breath metabolic carts to measure resting metabolic rate in young adults. *Nutrition, Metabolism and Cardiovascular Diseases* 2018 **28** 929–936. (doi:10.1016/j.numecd.2018.03.010)

4. Lucia A, Hoyos J, Perez M, & Chicharro JL. Heart rate and performance parameters in elite cyclists: A longitudinal study. *Medicine and Science in Sports and Exercise* 2000 **32** 1777–1782. (doi:10.1097/00005768-200010000-00018)

5. Fullmer S, Benson-Davies S, Earthman CP, Frankenfield DC, Gradwell E, Lee PSP, Piemonte T, & Trabulsi J. Evidence Analysis Library Review of Best Practices for Performing Indirect Calorimetry in Healthy and Non-Critically Ill Individuals. *Journal of the Academy of Nutrition and Dietetics* 2015 **115** 1417-1446.e2. (doi:10.1016/j.jand.2015.04.003)

6. Weir JB de V. New methods for calculating metabolic rate with special reference to protein metabolism. *The Journal of Physiology* 1949 **109** 1–9.

7. Frayn KN. Calculation of substrate oxidation rates in vivo from gaseous exchange. *Journal of applied physiology: respiratory, environmental and exercise physiology* 1983 **55** 628–634. (doi:10.1152/jappl.1983.55.2.628)

8. Buysse DJ, Reynolds CF, Monk TH, Berman SR, & Kupfer DJ. The Pittsburgh sleep quality index: A new instrument for psychiatric practice and research. *Psychiatry Research* 1989 **28** 193–213. (doi:10.1016/0165-1781(89)90047-4)

9. Communications S. Nutrition and Athletic Performance. *Medicine and Science in Sports and Exercise* 2016 **48** 543–568. (doi:10.1249/MSS.0000000000000852)

10. Rollo I, Gonzalez JT, Fuchs CJ, Loon LJC van, & Williams C. Primary, Secondary, and Tertiary Effects of Carbohydrate Ingestion During Exercise. *Sports Medicine* 2020 **50** 1863–1871. (doi:10.1007/s40279-020-01343-3)

**SUPPLEMENTARY RESULTS**

**Table S1.** Dietary supplements and ergogenic aids nutritional information

| **Evowhey 2.0 (WPC whey protein concéntrate) (100g)** | Energy  Total fat  Saturated fat  Total carbohydrates  Total sugars  Protein  Salt | 417kcal  6.8g  4.7g  7.9g  5.8g  81g  1g |
| --- | --- | --- |
| **Evogummy recovery (1 bar – 30g)** | Energy  Total fat  Total carbohydrates  Total sugars  Dietary fiber  Protein  Salt | 30g/bar  0.1g  20g  16.2g  0.9g  0.4g  0.1g |
| **Evoenergy sport gel with guarana and caffeine (1 gel- 50g)** | Energy  Total fat  Total carbohydrates  Total sugars  Protein  Caffeine  Guarana  Sodium  Potassium  Vitamin C | 124kcal  0g  31g  17g  0g  75mg  100mg  60mg  50mg  80mg |
| **Evoenergy without caffeine sport gel (1 gel – 50g)** | Energy  Total fat  Total carbohydrates  Total sugars  Protein  Sodium  Potassium | 124kcal  0g  31g  17g  0g  60mg  50mg |
| **Evocarbs 2.0 (40g – 2 dispensers)** | Energy  Total fat  Saturated fat  Total carbohydrates  Total sugars  Protein  Salt  Sodium | 141kcal  0g  0g  35g  16g  0g  0.79g  311.7mg |
| **Evolytes (1 capsule)** | Calcium  Magnesium  Sodium  Potassium  Chloride | 10mg  7.3mg  249.5mg  60.3mg  439mg |
| **Creatine monohydrate powder (100% Creapure)** | 100% Creatine monohydrate |  |
| **Anhydrous caffeine tablet (1 tablet)** | Caffeine  Pantothenic acid  Vitamin B6 | 200mg  6mg  0.7mg |
| **Beta-Alanine powder 100% RAW** | 100% Beta-Alanine |  |
| **Chelated Iron (Ferrous bisglycinate and ferrous sulphate) (1 capsule)** | Iron | 25mg |

All dietary supplements and ergogenic aids were provided by Harrison Sport Nutrition (HSN^®^) Store, Granada, Spain.


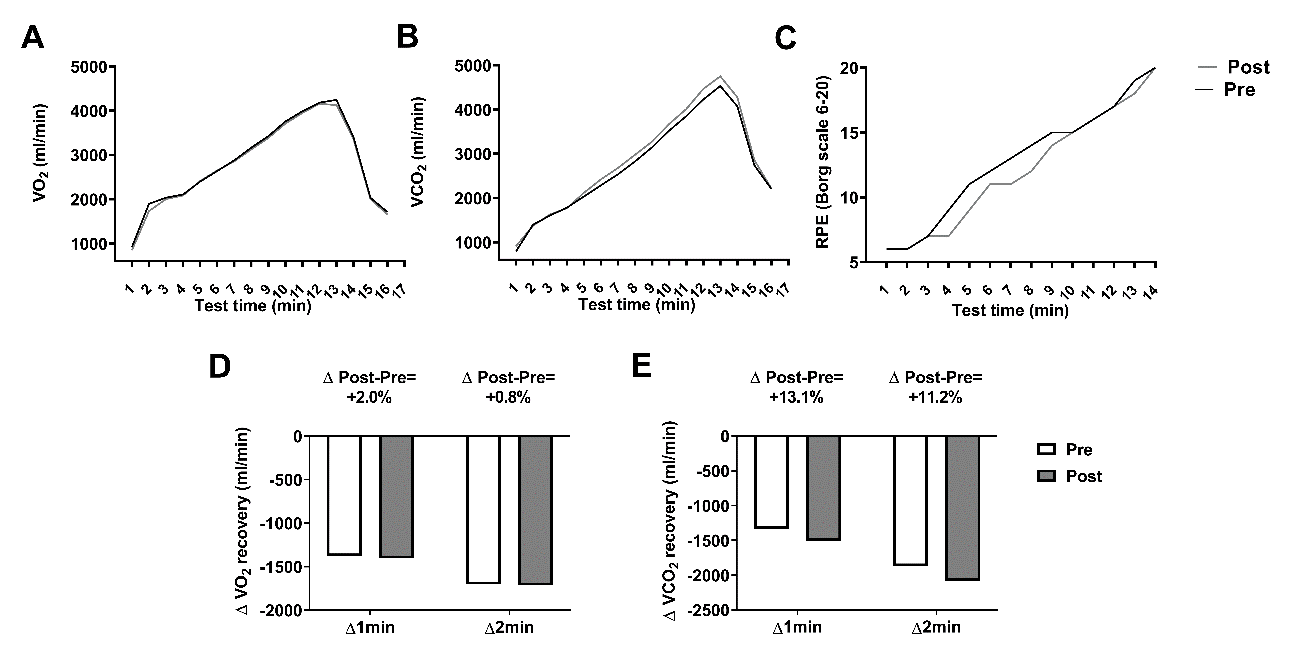


**Figure S1. Changes in the kinetics of the VO_2_, VCO_2_ and RPE derived from the maximal effort treadmill test (Panels A-C) and the VO_2_, and VCO_2_ recovery after the maximal effort treadmill test (Panels D and E).** Δ1min was calculated as: Recovery value minute 2-value recovery minute 1; whereas Δ2min was calculated as: value recovery minute 3-value recovery minute 1. *Abbreviations*: RPE, rate of perceived exertion; VCO_2_, volume of carbon dioxide production; VO_2_, volume of oxygen consumption.
